# OA28 A rare case of cardiac tumour in infancy mimicking sarcoidosis

**DOI:** 10.1093/rap/rkac066.028

**Published:** 2022-09-28

**Authors:** Ayesha Al Mheiri, Kirsty McLellan, Alice Burleigh, Ebun Omoyinmi, Fiona Price-Kuehne, Paul Brogan

**Affiliations:** Great Ormond Street Hospital, Rheumatology Department, London, United Kingdom; Great Ormond Street Hospital, Rheumatology Department, London, United Kingdom; University College London, Institute of Child Health, London, United Kingdom; University College London, Institute of Child Health, London, United Kingdom; University College London, Institute of Child Health, London, United Kingdom; Great Ormond Street Hospital, Rheumatology Department, London, United Kingdom; University College London, Institute of Child Health, London, United Kingdom

## Abstract

**Introduction/Background:**

Sarcoidosis is a rare multisystemic inflammatory disorder with 2 distinct presentations in childhood. A monogenic form associated with mono-allelic mutation in *NOD2* presents with the triad of uveitis, rash and arthritis in children less than 4-years (early-onset sarcoidosis). The other presentation affects older children with similar presentation to adults, with greater pulmonary and lymph node involvement. Histopathological evidence of non-caseating granulomas is essential for the diagnosis but does not exclude alternative diagnoses. We report a 17-month-old infant, who presented with atypical features suggestive of sarcoidosis, but was subsequently found to have a rare cardiac angiosarcoma driving an exaggerated granulomatous reaction.

**Description/Method:**

A previously healthy 17-month-old girl born to non-consanguineous Caucasian parents presented with recurrent fever for 3-months, associated with lethargy, weight loss and worsening anemia. Examination revealed an irritable febrile child, weight on 5th centile, with muffled heart sounds; there were no skin lesions, no lymphadenopathy, and no arthritis. Investigations showed severe anemia (Hb 46g/L), leukocytosis (WBC 24/L), thrombocytosis (662/L), high inflammatory markers (CRP >300mg/L, ESR 160mm/hr), high ferritin (600ug/L). CT thorax with cardiac angiogram revealed a large intra-atrial septal mass, extensive pulmonary nodules, bilateral hilar and mediastinal lymphadenopathy and pericardial effusion. She was managed with broad-spectrum antibiotics, blood transfusions and pericardiocentesis with endomyocardial biopsy. Extensive infectious workup, including TB from pericardial exudate, was negative. Serum angiotensin-converting enzyme was normal, with no blasts in blood film; eye examination was unremarkable. The endomyocardial biopsy was inconclusive, so underwent lung and lymph node biopsies, which were initially reported as revealing florid multiple non-necrotising granulomas, but no evidence of malignant cells. Whole-exome sequencing was performed, result pending at that stage. Differential diagnosis included malignancy, infection, immunodeficiency or autoimmune disorder including sarcoidosis, in light of histopathology. With malignancy and infection felt to be excluded, she was managed empirically for atypical sarcoidosis and treated with IV methylprednisolone 30mg/kg followed by tapering steroid and anti-TNF; IL-1 was added later. However, there was no clinical response with ongoing fever, irritability and static inflammatory markers (CRP∼300mg/L). Immunomodulating medications were subsequently stopped. Repeat chest imaging revealed progression of pulmonary findings and increasing cardiac mass. Open-heart bypass surgery with debulking of cardiac mass and further biopsies. Histopathology revealed an extremely rare primary cardiac malignant vascular tumour, angiosarcoma, with loss of SMARCB1 protein staining. Retrospective review and specific staining with SMARCB1 of initial lung biopsy subsequently confirmed that the granulomatous reaction was in response to micro-metastases of tumour throughout the lungs.

**Discussion/Results:**

The differential diagnosis of granulomatous disease in paediatric patients is very broad. Ultimately, the diagnosis of cancer driving and exaggerated immunological response was provided by a combination of very invasive surgical biopsy of the heart; combined with whole exome sequencing. Whole exome sequencing revealed 2 distinct genetic diagnoses which fully explained the phenotype: heterozygosity for a stop mutation in *SMARCB1* (p.R158X) consistent with Rhabdoid Tumour Predisposition Syndrome Type 1; and homozygosity for the hypomorphic *PRF1* p.A91V mutation explaining low (but not absent) intracellular perforin levels and exaggerated granulomatous immunological response to tumour. The rare angiosarcoma detected is scarcely reported in the literature, but entirely compatible with the type of cancer associated with RTPS1. Hypomorphic perforin mutation probably explains the exaggerated but frustrated granulomatous immunological response to tumour in this case.

**Key learning points/Conclusion:**

This case serves to remind us that granulomas can occur in response to malignancy in children. Lack of response to immunosuppression with ongoing severe systemic inflammation in this case provided an important clue to the need for more invasive diagnostic biopsies. Particular challenges relates to the time it takes to obtain genetic test results; but in this case whole-exome sequencing resolved the phenotype fully. Patients with rare and unusual presentations may have more than one genetic diagnosis, with a blending of phenotypes thereafter. Screening of other family members for the mutation associated with RTPS1 is ongoing.

